# 肿瘤靶向治疗和免疫治疗——眼科系统毒性反应的诊治

**DOI:** 10.3779/j.issn.1009-3419.2019.10.09

**Published:** 2019-10-20

**Authors:** 小伟 刘, 铮 王, 潺 赵, 汉萍 王, 潇潇 郭, 佳鑫 周, 炼 段, 晓燕 斯, 丽 张, 玥 李, 孟昭 王, 举红 施, 美芬 张, 力 张

**Affiliations:** 1 100730 北京，中国医学科学院，北京协和医院眼科 Department of Ophthalmology, Peking Union Medical College Hospital, Peking Union Medical College and Chinese Academy of Medical Sciences, Beijing 100730, China; 2 100730 北京，北京医院眼科 Department of Ophthalmology, Beijing Hospital, Beijing 100730, China; 3 100730 北京，中国医学科学院，北京协和医院呼吸内科 Department of Respiratory Medicine, Peking Union Medical College and Chinese Academy of Medical Sciences, Beijing 100730, China; 4 100730 北京，中国医学科学院，北京协和医院心内科 Department of Cardiology, Peking Union Medical College and Chinese Academy of Medical Sciences, Beijing 100730, China; 5 100730 北京，中国医学科学院，北京协和医院风湿免疫科 Department of Rheumatology, Peking Union Medical College and Chinese Academy of Medical Sciences, Beijing 100730, China; 6 100730 北京，中国医学科学院，北京协和医院内分泌科 Department of Endocrinology, Peking Union Medical College and Chinese Academy of Medical Sciences, Beijing 100730, China; 7 100730 北京，中国医学科学院，北京协和医院检验科 Department of Clinical Laboratory, Peking Union Medical College and Chinese Academy of Medical Sciences, Beijing 100730, China; 8 100730 北京，中国医学科学院，北京协和医院消化内科 Department of Gastroenterology, Peking Union Medical College Hospital, Peking Union Medical College and Chinese Academy of Medical Sciences, Beijing 100730, China

**Keywords:** 靶向治疗, 免疫检查点抑制剂, 免疫相关不良反应, 眼部疾病, Target therapy, Immune checkpoint inhibitor, Immunotherapy-related toxicities, Ocular diseses

## Abstract

靶向治疗和免疫治疗给晚期肿瘤患者带来了希望。但是治疗过程中的药物毒副作用也随着药物的普及而逐渐展现出来。其中眼部的毒副反应常被患者和医生忽视。眼部的不良反应包括从眼睑、睫毛、结膜、角膜、葡萄膜、视网膜、视神经和眼眶等，可累及眼部的所有组织。本文就靶向治疗和免疫治疗相关的眼部毒副作用的诊断和治疗给予相应的描述和建议。

## Abstract (en)

The increasing use of target therapy and immunocheckpoint inhibitors in cancers has brought new hope of survival to patients with advanced tumors. However, more and more adverse side-effects and toxicities of these medications had been reported, affect almost all human organs including the eye. These adverse effects may affect the entire ocular tissues, like eyelid, eye lashes, conjunctiva, cornea, uvea, retina, optic nerve and so on, which are always been ignored by patients and doctors. In this paper we will summarize the characteristics of the related ocular diseases and give our advice on how to diagnose and manage these diseases.

靶向治疗和免疫治疗已成为恶性肿瘤患者，尤其是全身转移患者的最后救命稻草。前者是定向杀灭肿瘤细胞，后者是通过上调机体T细胞对肿瘤细胞的杀伤作用而控制肿瘤生长。这些新的治疗方法能够在一定程度上延长患者的寿命，控制肿瘤的发展；然而随之而来的不良反应也逐渐被人们所认知。其中眼部的不良反应相对于全身反应来说相对较轻，但是却严重威胁患者的视力和生活质量，应当引起充分重视。目前医生和患者对其了解和重视程度还很不够，这主要是因为多数患者全身情况较差，即使出现一些不太严重的眼部的毒副作用，也经常被医生和患者忽略；此外，多数肿瘤患者已给家人精力和经济带来严重的负担，为了避免加重相关负担，患者多选择忽略眼部的不良作用。因此，肿瘤医生了解和预知可能出现的毒副作用，及时转诊眼科医生，则尤为重要和必要。

从早期的一期或二期临床试验报告中，我们常可以查到视物模糊、眼部不适等不良反应^[1]^，但是并不能获得导致具体眼部病变的类型和临床特点。其后陆续有个案文献报道眼部的毒性反应包括：睑炎、结膜炎、葡萄膜炎^[2-4]^、巩膜炎^[5]^、脉络膜视网膜炎^[6]^等。然而其病理机制还不完全明确。其中细胞毒性T淋巴细胞相关抗原4（cytotoxic T lymphocyte associated antigen 4, CTLA4）单抗（ipilimumab）发生眼部不良反应的比例为1.3%^[7]^，包括前葡萄膜炎、视神经病变、Grave’s样病变、眼眶炎症和VKH样综合征^[8]^；Vemurafenib的眼部不良反应约4%，主要是葡萄膜炎^[8]^。炎症可持续数天到数年不等。葡萄膜炎多表现为双眼的轻度到中度的前或中间部葡萄膜炎。而免疫检查点分子程序性死亡受体-1（programmed cell death receptor-1, PD-1）单抗的主要眼部不良反应有视物模糊、流泪等^[1, 9]^。

本文就靶向治疗和免疫治疗中的作者临床遇到的和文献报道的眼部并发症进行详细介绍，并探讨其发生机理、检测和基本治疗原则。

## 1 眼睑皮肤和毛发改变

其发生的可能机制是药物抑制了眼部组织（包括睑板腺、毛囊、结膜、角膜、泪腺、眼睑皮肤和毛细血管）的表皮生长因子受体（epidermal growth factor receptor, EGFR）信号传导。

### 1.1 临床表现诊断和治疗

眼睑皮肤皮疹多见于肺癌靶向治疗患者，其皮疹与全身皮疹性质一样（图 1）。

**1:**
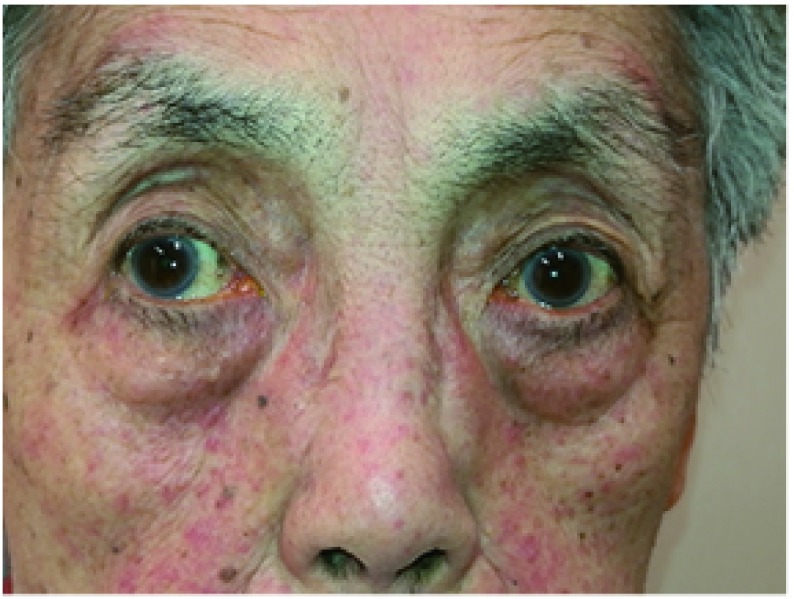
83岁女性，因肺癌口服易瑞沙治疗，面部及眼睑皮疹。同时可见睫毛和眉毛变粗变长变黑，且变长的睫毛常弯曲，打结。 Eighty-three years old female with lung cancer and treated with target therapy. This photo shows the erythema of face and eyelids. Trichomeglsy and eyebrow overgrow was also showed in this patient.

#### 1.1.1 症状和体征

面部和眼睑部较小红色皮疹、轻度突出于皮肤面，多无任何症状，部分患者有刺痒感和不适感觉。

#### 1.1.2 诊断

根据眼部皮肤表现，无须特殊检查可基本确诊。

#### 1.1.3 治疗

眼部皮疹多无症状，不需要处理，如果患者出现痒和刺激症状，可局部使用氧氟沙星眼膏或妥布霉素地塞米松眼膏减轻症状。

#### 1.1.4 预后

预后良好，多不需特殊治疗。

### 1.2 睫毛眉毛变长、变黑、增生也常见于EGFR靶向治疗患者。

#### 1.2.1 症状和体征

眉毛变粗、黑，变长且杂乱；睫毛亦可变长、变黑，但是睫毛末梢常弯曲并纠缠在一起（图 1）。甚至部分睫毛弯曲刺激角膜。

#### 1.2.2 诊断

根据典型的临床表现可确诊。

#### 1.2.3 治疗

因其不会引起不良反应，多不需治疗。部分患者因睫毛过长弯曲而刺激角膜，则需要修剪睫毛或给予拔除。

#### 1.2.4 预后

预后良好，多不需特殊治疗。

### 1.3 睑内翻或睑外翻亦偶有报道^[10]^，其发生原理尚不明确。

#### 1.3.1 症状

流泪和眼部异物感，分泌物增多。

#### 1.3.2 体征

眼睑内翻或外翻，结膜充血，分泌物增多，严重时可因结膜或内翻的皮肤摩擦角膜导致角膜上皮损伤、溃疡。

#### 1.3.3 诊断

根据典型的临床症状和体征可确诊。

#### 1.3.4 治疗

以手术治疗为主，矫正内翻或外翻。

#### 1.3.5 预后

预后良好，多不需特殊治疗。

## 2 睑缘炎和结膜炎

多见*EGFR*突变的靶向治疗^[10]^中，其发生机理可能与前述相同。

### 2.1 症状

眼痒、眼干并有异物感、烧灼感等。严重时会出现畏光、流泪症状，也可继发结膜炎，分泌物增多。

### 2.2 体征

睑缘充血、肥厚，可见睫毛根部皮屑或分泌物黏附。睑板腺开口充血、扩张，并有角化现象（图 2）。睫毛容易脱落，但可以再生。结膜多有充血、乳头增生。部分患者可表现为角膜表面点状的上皮缺损。

**2:**
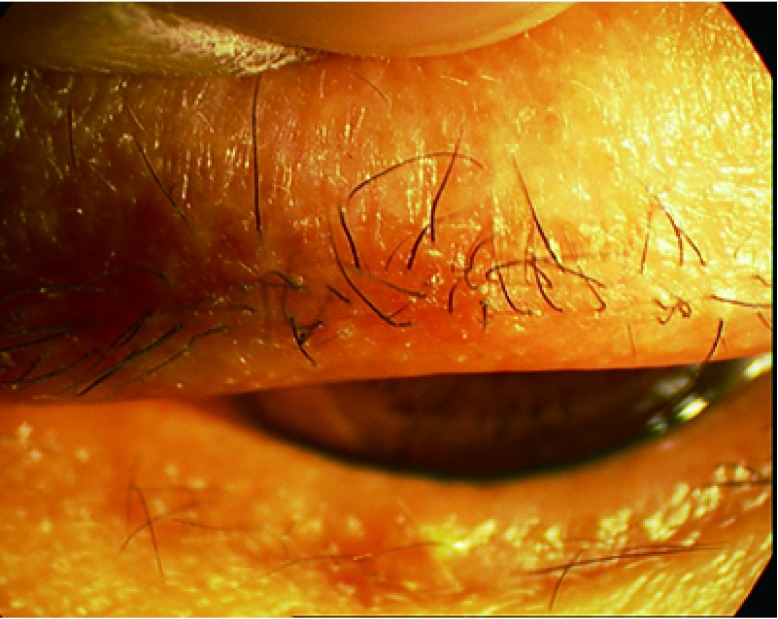
女性，71岁，肺癌晚期口服厄洛替尼1年，出现睑缘炎症，充血、肥厚。 The blepharitis of a 71-year old lung cancer patient treated with target therapy.

### 2.3 诊断

依据典型的症状和睑缘充血、肥厚，睫毛根部鳞屑附着可明确诊断。睑缘炎有鳞屑性睑缘炎、溃疡性睑缘炎和眦部睑缘炎三种，在靶向药治疗和免疫治疗相关不良反应（immune-related adverse effects, irAEs）中以鳞屑性睑缘炎比较常见。

### 2.4 治疗

① 热敷和清洗睑缘，改善局部卫生条件，热敷后睑板腺分泌物易于液化并顺利排出。②可用带有激素的眼膏（妥布霉素地塞米松眼膏）涂抹在睫毛根部，以减轻炎症反应，并起到润滑睑缘的作用。③眼部抗炎药：包括低浓度激素滴眼液和非甾体抗炎滴眼液的使用。④干眼症也是免疫治疗的一个主要的眼部并发症^[11]^。对于伴发干眼症患者，应给予人工泪液治疗，严重者需要进行泪小点栓塞手术。

### 2.5 预后

多不会引起严重的眼部并发症，给予对症治疗后多可缓解，但是停药或停止局部清洁后容易复发。

## 3 干眼症

其发生机制尚不十分明确，CTLA4和PD-1的临床试验文献中均有不同程度干眼症的发生，发生率为1.2%-24.2%^[11]^。但具体细节和数据均无描述。虽然其放生率较高，但是正常人群中干眼的发生率也比较高，因此常被忽视。

### 3.1 症状

干涩感、异物感、烧灼感、视力疲劳、眼胀、眼疼、畏光、眼红等。

### 3.2 体征

① 眼部充血，球结膜失去光泽；②泪河变窄；③角膜荧光染色可见角膜上皮点状或片状着色；④泪膜破裂时间变短（< 10 s）；⑤泪液分泌检查：泪液分泌减少，小于正常值（10 mm-15 mm/5 min）。

### 3.3 诊断

干眼症的诊断需要结合以下几个方面：①症状；②泪膜稳定性检查；③眼表上皮细胞的损伤多可做出诊断。

### 3.4 治疗

① 消除诱发干眼的诱因，减少手机和电脑屏的使用；②人工泪液，可补充泪液中水分的减少。目前临床常用的人工泪液包括：玻璃酸钠滴眼液、聚乙二醇滴眼液以及羧甲基纤维素等；③抗炎药物：多用低浓度糖皮质激素滴眼液和非甾体类抗炎药物；④泪小点栓塞，可减少泪液流出和蒸发，延长泪液在眼表的停留时间；⑤对于合并睑板腺功能障碍的患者，则需要加强睑缘清洁和局部热敷按摩增加睑板腺分泌物的排出。

### 3.5 预后

文献报道的靶向药和免疫治疗药物并未引起非常严重的干眼，未有因干眼导致严重视功能和眼部结构严重破坏的病例。经过对症治疗后预后良好，但是需要长期持续使用人工泪液。

## 4 角膜炎

角膜炎发生机理尚不十分明确，多见于肿瘤的靶向治疗中，可能是靶向或免疫治疗药物影响了角膜上皮创伤愈合的能力，导致角膜上皮持续缺损而致。

2009年Johnson等^[12]^报道了1例患者因使用Erlotinib导致可出现持续性的角膜上皮缺损、感染性角膜炎。经过治疗后角膜持续不愈合，直到停止使用Erlotinib之后，角膜溃疡才愈合，文献^[13, 14]^中角膜病变经，常规治疗难以奏效，需要中止靶向治疗，才能够使角膜病变愈合。

### 4.1 症状

怕光、流泪、眼红、视力下降。

### 4.2 体征

眼部充血（混合充血）、角膜上皮缺损、角膜浑浊，甚至出现溃疡。严重的患者可出现前房积脓，甚至角膜穿孔、眼球萎缩（图 3）。

**3:**
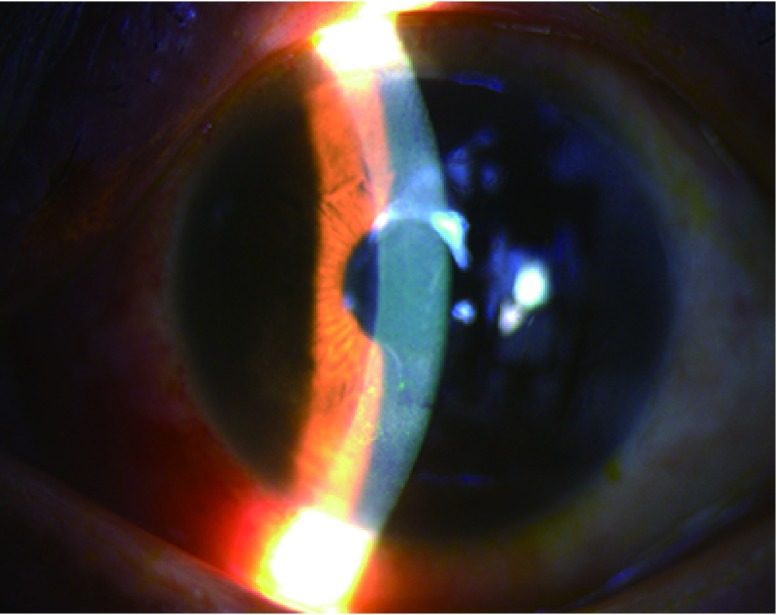
女性，49岁，因肺癌口服靶向药治疗导致角膜上皮病变 Keratopathy caused by target therapy in a 49-year old lung cancer patient

### 4.3 诊断

根据患者病史和眼部检查结果可基本确诊。

因肿瘤治疗药物造成的角膜炎并无显著的特征性，因此在确诊之前，一定要排除其他原因造成的角膜炎。比如外伤、干眼症或者病毒感染、细菌感染等造成的角膜炎等。当然，irAEs角膜炎可因继发感染而导致鉴别诊断更加困难。通细菌培养、细菌图片和角膜共焦显微镜检查等检查，可使我们为角膜炎的诊断和治疗提供有力的依据。

### 4.4 治疗

① 抗生素滴眼液预防继发感染；②使用促进角膜修复的药物：高浓度玻璃酸钠滴眼液、小牛血去蛋白提取物凝胶等；③严重者需要睫状肌麻痹剂；④绷带式角膜接触镜能够促进角膜的修复，但对于怀疑角膜感染的患者应当禁用；⑤对于角膜溃疡持续不愈合的患者，则要考虑中止靶向或免疫治疗。

### 4.5 预后

轻度患者愈合后常不会引起视力改变，而较严重的患者则可能导致严重的视力下降，甚至眼球丧失。

## 5 巩膜炎（包括浅层巩膜炎和巩膜炎）

巩膜炎的发生率不足1%^[15]^，可分为结节性巩膜炎和弥漫性巩膜炎。

### 5.1 症状

通常急性起病，表现为眼部疼痛、充血，轻度患者疼痛不明显，仅在按压时有疼痛感，疼痛可局限于眼球的某个部位。重度患者则全眼球甚至眶周疼痛剧烈，甚至头疼、恶心、呕吐。

### 5.2 体征

根据病变的部位和深度，将巩膜炎分类为前部巩膜炎、后部巩膜炎以及弥漫性巩膜炎；根据病变的深度和特点又分为：浅层巩膜炎和巩膜炎等。

浅层巩膜炎病变部位比较表浅，眼部充血呈鲜红色，而巩膜炎则病变部位较深，眼部充血呈紫红色。

前巩膜炎为眼球前部的巩膜炎症，多见片状或区域性浅层巩膜充血，血管扩张；结节性巩膜炎可在巩膜表面出现充血的结节病灶，多见于前巩膜炎；后巩膜炎和弥漫性巩膜炎则多出现弥漫性充血，伴随有明显的触痛和压痛，眼内炎症、视力下降以及全身症状。

严重的巩膜炎可引起视乳头水肿，后部脉络膜和视网膜水肿和炎症，从而导致视力下降。

### 5.3 辅助检查

① 超声检查：如果累及后部巩膜可发现后巩膜增厚，视神经轴位扫描因增厚水肿的巩膜与视神经回声相连而呈现" T" 征；②眼部磁共振成像（magnetic resonance imaging, MRI）检查亦可发现巩膜增厚。

### 5.4 诊断

根据局限性眼部充血、疼痛或触痛，而睑结膜无充血多可做出诊断。巩膜炎需要和结膜炎相鉴别：结膜炎时睑结膜和球结膜同时充血，而巩膜炎多为球结膜充血，而睑结膜无充血。必要时可以使用0.1%肾上腺素滴眼，可使结膜血管收缩，而巩膜血管则不能收缩，从而鉴别巩膜炎和结膜炎。对于弥漫性巩膜炎或后巩膜炎多有合并葡萄膜炎，则需要眼部B超或眼眶MRI检查以确诊。

### 5.5 治疗

① 浅层和前部巩膜炎，一般局部激素治疗即可，全巩膜炎和后巩膜炎则需要口服或静脉给予糖皮质激素治疗或其他免疫抑制剂。常用局部糖皮质激素包括：0.1%地塞米松滴眼液、1%泼尼松龙滴眼液、0.1%氟米龙滴眼液、0.5%氯替泼诺滴眼液等。其中0.1%地塞米松滴眼液和1%泼尼松龙滴眼液抗炎效果较强，但是局部长期使用可引起激素性青光眼或白内障等并发症，因此需要定期眼科检查。当局部炎症控制后可酌情减少点药次数和更换抗炎效果较弱的糖皮质激素如：氟米龙和氯替泼诺等。待炎症完全稳定后仍需继续用药1周-2周左右，巩固治疗效果；②局部或口服非甾体类抗炎药物：双氯芬酸钠滴眼液、口服吲哚美辛等；③全身使用免疫抑制剂：环孢霉素或环磷酰胺等。

### 5.6 预后

多数患者预后良好，对局部糖皮质激素反应良好，但是严重巩膜炎一旦延误治疗，有可能导致永久性视力丧失。

## 6 葡萄膜炎

虹膜、睫状体和脉络膜统称为葡萄膜，葡萄膜富含色素细胞和血管，因此是眼部极易发生自身免疫性疾病的组织。CTLA4和PD-1单抗因能够上调T细胞的功能，增加自身免疫疾病的发生而常引起葡萄膜的炎症^[16]^；而在一项肿瘤浸润淋巴细胞（tumorinfiltratinglymphoeytes, TILs）+IL-2的临床研究中，35例患者中，亦有5例发生了葡萄膜炎，通过局部激素均可控制症状^[17]^。多数症状发生于用药后12周内，治疗及时可在6周-8周内缓解^[8]^。

葡萄膜炎可分为前部葡萄膜炎、中间部葡萄膜炎和后部葡萄膜炎以及全葡萄膜炎。irAEs引起的葡萄膜炎多种多样，各种类型均有^[16]^。

### 6.1 症状

① 眼部疼痛；②怕光、流泪；③视力下降。

### 6.2 体征

① 睫状充血，局限于角膜缘的血管扩张；②角膜后沉淀物（keratic precipitates, KP），可有羊脂状和非羊脂状；③房水闪光和浮游细胞，血-房水屏障破坏，血管通透性增加导致细胞和蛋白成分渗漏到前房内，严重时可出现成形性渗出或前房积脓；④少数患者可能出现继发性眼压升高^[8]^；⑤虹膜纹理不清，充血，与晶状体黏连或与前房角黏连，从而导致瞳孔不规则改变（图 4）；⑥晶状体改变：色素沉着或并发白内障；⑦玻璃炎症，可尘状浑浊，团块状浑浊；⑧视网膜脉络膜炎症：包括视盘水肿、视网膜水肿、局灶或散在浸润灶，视网膜出血、渗出。视网膜血管变细、白鞘，黄斑水肿，视网膜渗出性脱离等改变；⑨视网膜、脉络膜血管炎^[8, 18]^。

**4:**
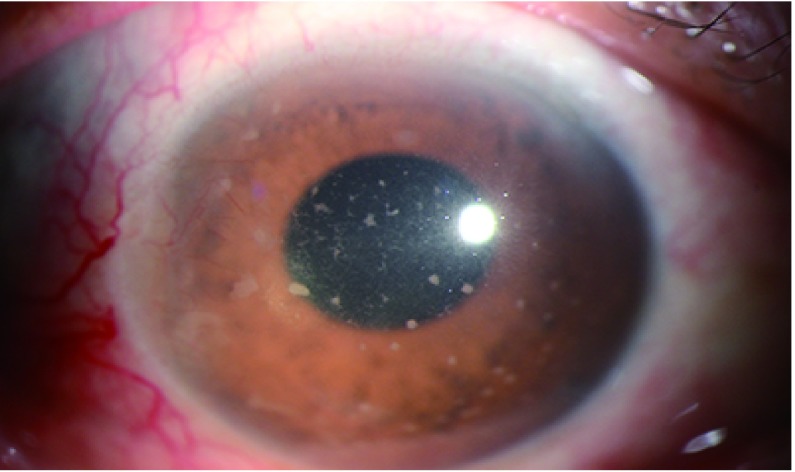
显示葡萄膜炎导致睫状充血和角膜后羊脂状KP Uveitis cause by immunocheckpoint inhibitors, the white dot in the cornea were keratic precipitates

### 6.3 辅助检查

① 眼部超声：可了解玻璃体炎症和视网膜脉络膜情况；②视网膜和脉络膜血管造影：有助于评估视网膜脉络膜血管病变；③光学相干断层扫描（optical coherence tomography, OCT）：对于视网膜结构改变和视网膜下渗出性脱离均有重要的作用。

### 6.4 诊断

根据典型的临床表现和眼部体征即可做出初步诊断。①临床：根据眼部疼痛、视力下降、畏光等症状，和眼部充血（以角膜周围血管扩张为主——睫状充血）、角膜后沉淀物（KP），房水闪光和瞳孔缩小、虹膜后粘连等即可诊断。对于累及玻璃体和视网膜的葡萄膜炎则可见玻璃体混浊和视乳头、视网膜水肿、血管炎、黄斑水肿等改变；②影像学诊断：眼底荧光血管造影（fundus fluorescence angiography, FFA）和吲哚箐绿脉络膜造影（indocyanine green angiography, ICGA）、眼部超声、OCT、电子计算机断层扫描(Computed Tomography, CT)、磁共振成像（Magnetic Resonance Imaging, MRI）等影像学技术能够帮助诊断；③前房水内细胞因子检测，也有助于特殊类型的葡萄膜炎的诊断。

### 6.5 治疗

① 散瞳：解除睫状肌痉挛，缓解疼痛，同时治疗和防止瞳孔黏连；②糖皮质激素：局部或全身使用糖皮质激素，是治疗免疫治疗引起葡萄膜炎的主要手段，且反应良好^[8, 19]^。一般irAEs引起的葡萄膜炎多需要全身使用糖皮质激素，多从1 mg/kg体质量开始，根据眼部炎症情况，逐渐减量，疗程多需要3个月以上。除滴眼液和口服外，曲安奈德球侧注射^[16]^有时也是一种有效的治疗手段；③免疫抑制剂：对于糖皮质激素疗效不佳或禁忌者，可考虑使用免疫抑制剂与糖皮质激素联合使用，比如：环磷酰胺或硫唑嘌呤等；④非甾体类抗炎药；⑤其他治疗：如眼底激光等。

### 6.6 预后

多数葡萄膜炎对糖皮质激素反应良好，可快速控制炎症。对于难治性葡萄膜炎，则可能需要眼科医生与肿瘤科医生协商利弊是否停用免疫治疗。

## 7 Vogt-Koyanagi-Harada（VKH）综合征样改变

VKH综合征，又称为小柳原田病综合征，是葡萄膜炎的一种特殊类型，又称为特发性葡萄膜大脑炎：其主要特征是弥漫性渗出性葡萄膜炎伴有全身性的脑膜刺激症状、听力障碍、白癜风、毛发变白和脱落等。

因其目前是免疫治疗中最为常见和严重的眼部不良反应，因此本文中单独给与描述。VKH综合征是一组由T淋巴细胞介导的，针对黑色素细胞的自身免疫反应症候。VKH的发生也与*CTLA4*基因的遗传多态性也有一定有关系。CTLA4和PD-1单抗通过上调T细胞的功能，刺激CD4和CD8T细胞针对宿主和肿瘤细胞的黑色素细胞抗原进行攻击从而导致肿瘤特异性免疫和自身免疫平衡状态的破坏。因此，CTLA4和PD-1单抗治疗中引起的VKH样综合征发生率相对较高。

CTLA4^[20]^和PD-1单抗单独使用^[21, 22]^或二者联合^[23, 24]^应用均有引起VKH样病变的报道，TIL联合IL-2治疗也有类似报道^[17]^。其原发肿瘤多见于转移性黑色素瘤^[22]^，也有非小细胞肺癌的报道^[21]^。

### 7.1 症状

① 前驱症状：发热、头疼、耳鸣、听力下降、颈项强直、颅压增高；②视力下降；③神经系统：头疼、头晕、恶心和颈项强直等脑膜刺激症状；④听力：耳鸣、听力下降；⑤皮肤：白癜风、白发、脱发（图 5）。

**5:**
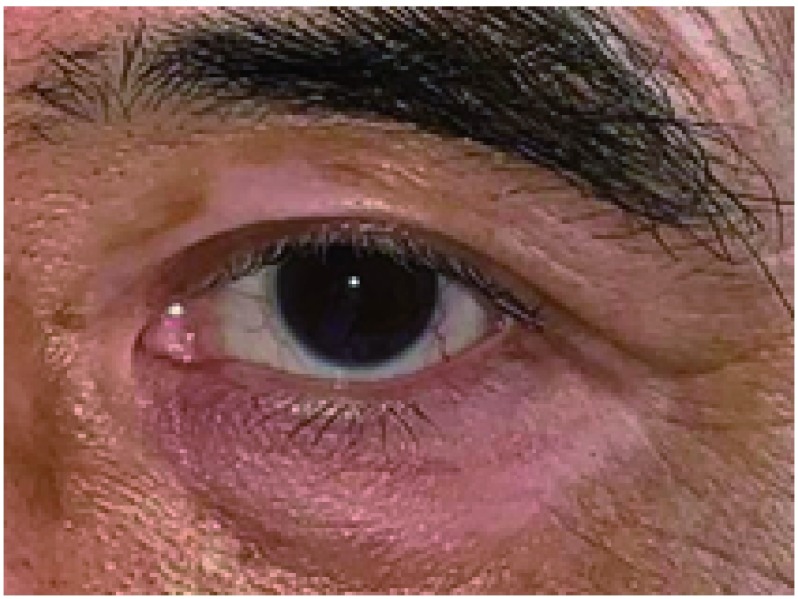
患者眼周皮肤白癜风，睫毛变白 Eyelid skin vitiligo and poliosis of th eyelashes in a VKH patient

### 7.2 体征

① 眼部充血；②角膜后羊脂状沉淀物；③前房闪辉、渗出，可见浮游细胞；④瞳孔黏连；⑤玻璃体浑浊；⑥视盘充血、水肿；⑦浆液性视网膜脱离；⑧晚期视网膜色素萎缩呈现晚霞状眼底，亦可见白色结节样病灶（图 6和图 7）。

**6:**
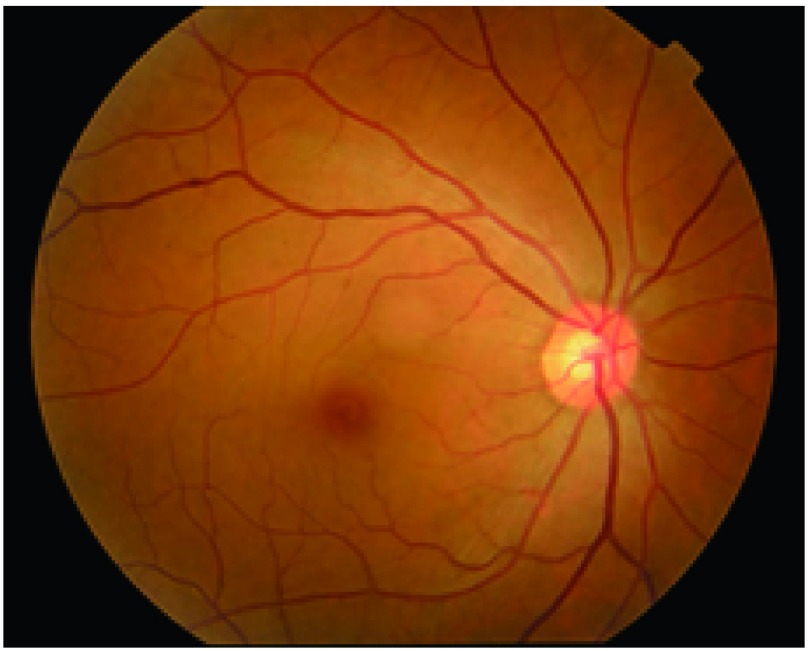
正常眼底呈橘红色 Normal fundus

**7:**
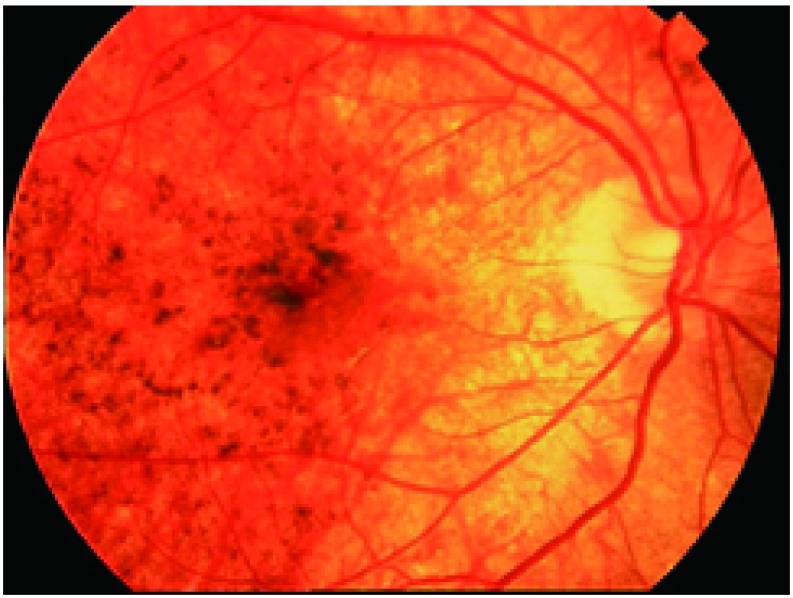
晚霞状眼底 "sunset glow fundus"in a VKH patient

与典型VKH相比，免疫治疗中VKH样改变变异较大：可表现为完全相同，包括前驱症状和各种症状出现的顺序^[24]^；也可出现为不典型临床表现^[17]^。①VKH表现不完全：比如缺乏脑膜刺激征、听力障碍、白癜风等^[18]^；②时间顺序可能不同：可能不出现前驱症状，无发热，或先于视网膜并变前出现其他部位色素改变白癜风等^[17, 22]^；③症状轻重不一，部分患者无任何眼前节炎症反应，而眼部仅出现黄斑区浆液性视网膜脱离^[23]^。而有些患者则表现为非常严重的前节反应，也可发现时已经出现晚期的晚霞状眼底，而无浆液性渗出性视网膜脱离表现^[25]^；④对糖皮质激素反应差异较大，有些患者反复发作；⑤用药后出现眼部症状的时间跨度很大（2周-13个月不等）。

### 7.3 诊断

根据典型的临床表现和眼部体征即可确诊。

眼底OCT或FFA可发现多发性的浆液性视网膜脱离（图 8）。然而多发浆液性视网膜脱离也可见于脉络膜转移癌。尤其是肺癌眼部转移，是一个非常常见的转移部位，有时会导致诊断的困难，需要进行详细的检查和鉴别（图 9，图 10）。

**8:**
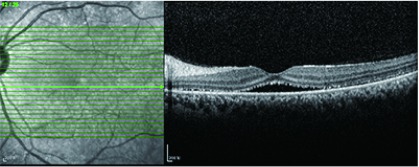
浆液性视网膜脱离 Retinal serum detachment of the central retina in a PD1 treated patient

**9:**
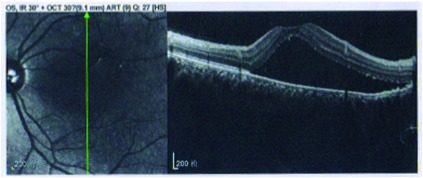
39岁肺癌女性，PD-1单抗治疗中，左眼视力下降，OCT显示为多发浆液性视网膜脱离，误诊为VKH样改变，经激素治疗无效，最终确诊为肺癌眼部转移。 Retinal serum detachment in a 39-year old female with advanced lung cancer, who was treating with PD-1 antibody. She complained vision loss of the left eye and misdiagnosed with VKH syndrome induced by PD1, but didn't response to oral corticosteroid. Finally, her left eye was proved to be ocular metastatic tumor.

**10:**
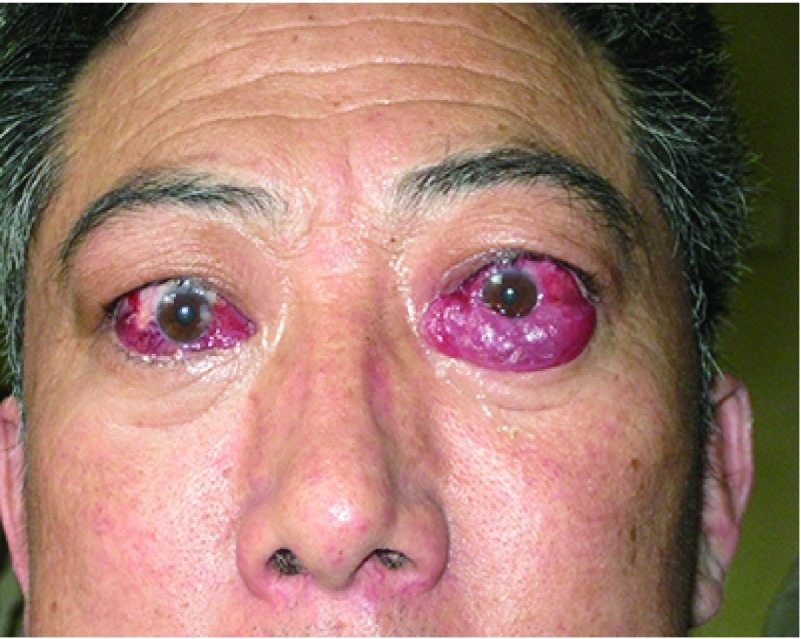
甲状腺相关性眼病 Thyroid associate orbitopathy

脑脊液检查可发现：淋巴细胞增多。

### 7.4 治疗

同葡萄膜炎。

### 7.5 预后

预后取决于患者对糖皮质激素的反应，多数患者预后良好，少数严重患者需要中止免疫治疗，有导致肿瘤复发的可能。因此需要眼科医生和肿瘤医生以及患者共同合作，才能制定最佳的治疗方案。

## 8 Grave’s眼病样改变

Grave’s眼病也称为甲状腺相关性眼眶病变，其发病机制与T细胞免疫有关。因此CTLA4单抗既可导致甲状腺机能亢进，同样也可导致眼眶病相关的自身免疫^[23]^，与患者眼眶内组织促甲状腺素受体异常表达有关。

### 8.1 症状

① 眼球突出、肿胀：眼眶内眼外肌增粗，软组织水肿和脂肪含量增加从而导致眼球突出。多为双侧性，亦可表现为单侧。②眼球疼痛，尤其是转动时疼痛明显。③眼红：常有结膜充血、水肿造成。④复视和眼球运动障碍：早期多因眼外肌炎症和水肿造成，晚期眼外肌纤维化而导致限制性斜视。眼球运动受限。⑤视力下降：当肥厚的眼外肌或眶内组织压迫视神经则会造成视力下降。

### 8.2 体征

① 眼睑：上睑退缩，迟落，导致睑裂过大，呈现惊恐状外观。有时也可表现为上睑下垂、下睑内翻等。②球结膜水肿：与炎症程度相关。③眼球突出：因眶内软组织过多造成。④斜视和复视，眼球运动障碍：多因眼外肌纤维化造成。⑤结膜角膜病变：结膜水肿，或因眼球突出，眼睑退缩导致眼睑闭合不全，从而导致结膜和角膜病变。⑥视神经病变：眶内压力增高可导致视神经受压。

### 8.3 辅助检查

① B超：可检查眶内脂肪和眼外肌肥厚情况；②眼眶CT、MRI：眼外肌肥厚；③实验室检查：甲功和甲状腺相关抗体可能会有异常发现。

### 8.4 诊断

根据典型的临床表现和体征，多可做出诊断。眼部超声、CT，MRI可发现眼外肌肥厚，且肥厚以肌腹肥大为主。

### 8.5 治疗

① 控制甲功的情况下，进行全身和局部糖皮质激素治疗；②当出现恶性突眼视神经受压时，药物治疗无效的情况下需要进行眶减压手术；③对暴露性角膜炎的治疗，其治疗原则是减少暴露、润滑眼表和促进眼表修复；④稳定期进行斜视和眼睑退缩的矫正手术和眶减压手术；⑤药物治疗无效或有禁忌者，可采用放疗。

### 8.6 预后

Graves眼病一般预后相对良好，对激素敏感患者多可控制急性期症状，稳定期主要针对并发症进行治疗。

## 9 眼部水肿和黄斑囊样水肿

多见于Imatinib，因其能够抑制眼眶周围c-Kit和血小板源性生长因子受体络氨酸激酶，从而导致毛细血管通透性增加，液体聚集。水肿可表现在眼睑周围，也可表现于眼底视网膜^[10]^。

## 10 渗出性视网膜脱离

抗血管内皮生长因子（vascular endothelial growth factor, VEGF）药物虽然用于肿瘤的治疗，但目前其对老年黄斑变性和黄斑水肿的治疗在眼科的应用似乎远超过在肿瘤中的应用。然而，全身使用抗VEGF药物却可导致渗出性视网膜脱离^[26]^和其可能引起血压升高而导致的视网膜动脉和静脉阻塞的风险增高^[10]^。其作用机理可能不是通过VEGF受体起作用，而是通过其他通过起作用^[26]^。

## 11 脉络膜新生血管膜的形成

2013年Modjtahedi等^[27]^报道了1例81岁的男性患者，因转移性黑色素瘤接受ipilimumab治疗，1年后患者出现双眼视力下降，检查提示脉络膜新生血管膜的形成和双侧黄斑水肿，经玻璃体腔注射雷珠单抗治疗后好转。

## 12 其他较少见的并发症

如：上睑下垂、眼肌麻痹等^[28]^，出现眼睑不能上抬至正常位置和眼球运动障碍、复视；此外视力波动、白内障、色觉异常、视觉干扰、偏盲、流泪、颞动脉炎等也时有报道^[9, 10, 29]^。

综上所述，靶向治疗和免疫治疗导致的眼部不良反应通常经局部或全身糖皮质激素即可控制或痊愈。而原有的免疫治疗一般不需要停止，对于严重的葡萄膜炎，患者出现严重的眼部疼痛和失明，则需要停止免疫治，但是并不影响患者的最终生存率^[30]^。停止使用免疫治疗后，眼部炎症多能自行好转。然后，停药还是继续使用免疫抑制或靶向药的选择却存在很大争议^[20]^。重新治疗可能会导致眼部病变的重新出现^[26]^，因此要谨慎开始，密切观察。

众多靶向药物和免疫治疗药物均可引起眼部的毒副作用，建议开始治疗前患者最好能够接受常规的眼部检查，包括视力、眼压、裂隙灯和眼底检查、眼底照相、视野和OCT检查。以便在发生眼部不良事件时能够明确是否为药物所引起的，还是之前就存在的病变。肿瘤科医生与眼科医生的密切合作，才能共同呵护肿瘤患者的健康和改善患者的生活质量。
